# Multi-omics integration identifies NK cell dysregulation and a five-gene diagnostic signature in major depressive disorder

**DOI:** 10.3389/fimmu.2025.1700629

**Published:** 2026-01-12

**Authors:** Jia Wang, Ye Kuang, Chuanmei Peng, Yong Yuan, Yong Ji, Sulian Chen, Jinrong Tian, Yuanyuan Zhou, Xingying Chen, Jing Li, Lei Feng, Shengjie Nie

**Affiliations:** 1 School of Forensic Medicine, Kunming Medical University, Kunming, China; 2 Department of Medical Laboratory, Yan’an Hospital Affiliated to Kunming Medical University, Kunming, China; 3 NHC Key Laboratory of Drug Addiction Medicine, Kunming Medical University, Kunming, Yunnan, China

**Keywords:** key genes, major depressive disorder, molecular mechanism, multi-omics integration, NK cells

## Abstract

**Background:**

Major Depressive Disorder (MDD), a leading global disability affecting 280 million people, has poor treatment efficacy due to persistent biological variability involving cell-type-specific transcriptomic dysregulation and immune dysfunction, and integrated multi-omics approaches are vital to uncover pathways and therapeutic targets.

**Methods:**

This research utilized a comprehensive multi-omics approach, merging bulk RNA sequencing (RNA-seq) data from the GSE39653 dataset with single-cell RNA sequencing (scRNA-seq) data derived from peripheral blood mononuclear cells (PBMCs) of three MDD patients and three healthy controls. Analysis of differential gene expression (DEGs1) and identification of genes inside Weighted Gene Co-expression Network Analysis (WGCNA) modules were conducted using bulk RNA-seq data. Analysis of differential cell population abundance and differential gene expression (DEGs2) was performed on the scRNA-seq data. Detection of CD3^-^CD56^+^ or CD3^-^CD16^+^ NK cells in human peripheral blood samples by flow cytometry. Candidate genes were subsequently identified from the intersection of DEGs1, WGCNA module genes, and DEGs2. Subsequently, machine learning methods were employed to discern key genes from these candidates. The functional characterization of essential cell populations was accomplished via pseudotime trajectory analysis, Gene Set Variation Analysis (GSVA), metabolic pathway analysis (scMetabolism), and transcription factor inference (SCENIC). Ultimately, diagnostic models, regulatory networks, and compound screenings were developed based on the key genes.

**Results:**

In the RNA-seq analysis, 803 DEGs1 and 2080 WGCNA module genes were identified. scRNA-seq analysis revealed 1,539 DEGs2 and identified natural killer (NK) cells as a major dysfunctional immune cell subpopulation in MDD, exhibiting a significantly increased proportion (CD3^-^CD56^+^ or CD3^-^CD16^+^, p < 0.05) in the MDD patients. The NK cell population was significantly enriched, flow cytometry validated this finding. The intersection of DEGs1, WGCNA module genes, and DEGs2 yielded 26 candidate genes. Subsequent machine learning analysis identified five key genes: *CSPP1*, *ZNF84*, *HLA-DPA1*, *CCZ1*, and *LRRC8D*. A diagnostic nomogram constructed using these key genes demonstrated robust discriminatory performance in distinguishing MDD patients. Mechanistic investigations implicated these five key genes in MDD pathogenesis through neurodegenerative signaling pathways.

**Conclusion:**

Our study establishes NK cell dysfunction as a core pathophysiological mechanism in MDD, characterized by cellular expansion and metabolic alterations. The identified key genes serve as robust diagnostic biomarkers and therapeutic targets. Elucidation of their regulatory networks provides critical insights for precision psychiatry interventions.

## 1 Introduction

Major depressive disorder (MDD) is a common and profoundly incapacitating mental illness. The primary clinical characteristics encompass a sustained sad mood or diminished interest, frequently associated with cognitive impairments and somatic symptoms, which considerably hinder patients’ social interactions, vocational efficacy, and overall quality of life (1). The current diagnosis of MDD predominantly depends on standardized criteria established in the American Psychiatric Association’s Diagnostic and Statistical Manual of Mental Disorders (DSM) or the World Health Organization’s International Classification of Diseases (ICD) (2). Epidemiological studies indicate that MDD is a leading cause of disability worldwide, affecting approximately 264 million individuals (3). In clinical practice, approximately 50% of patients receiving first-line antidepressant pharmacotherapy fail to achieve symptom remission, and nearly two-thirds require sequential treatment to attain the goal of clinical remission (4).

Contemporary treatment approaches for MDD predominantly rely on the monoamine neurotransmitter hypothesis. Traditional agents, including serotonin and norepinephrine reuptake inhibitors (SNRIs), selective serotonin reuptake inhibitors (SSRIs), tricyclic antidepressants (TCAs), and monoamine oxidase inhibitors (MAOIs), function by increasing monoamine neurotransmitter levels in the synaptic cleft. Nonetheless, these drugs demonstrate considerable limitations: around one-third of patients experience notable clinical enhancement, and a latency time of 3–4 weeks is generally necessary for little benefit to become apparent (5). Owing to the intricate pathophysiology of MDD, accurate diagnostic methods and efficacious pharmaceutical interventions are still constrained. Numerous hypotheses have been posited to elucidate the pathogenesis of MDD, including: (i) dysfunction of the hypothalamic-pituitary-adrenal (HPA) axis, (ii) deficiency of monoamines, (iii) inflammation, (iv) genetic and epigenetic anomalies, (v) structural and functional brain remodeling, and (vi) psychosocial factors. Nonetheless, none of these concepts independently elucidates the underlying foundation of MDD (6). This underscores the critical need to identify quantifiable biomarkers to enable precise diagnosis and stratified treatment.

Patients with MDD frequently exhibit features of immune dysregulation, including elevated levels of pro-inflammatory cytokines, altered immune cell function, and activation of inflammatory responses (7, 8). Levels of pro-inflammatory cytokines such as interleukin-6 (IL-6), tumor necrosis factor-alpha (TNF-α), and C-reactive protein (CRP) are typically elevated in individuals with MDD (9–11). These cytokines can access the central nervous system, influencing neurotransmitter metabolism, neuroendocrine function, and neuroplasticity, thereby contributing to the development of depressive symptoms (12). Furthermore, alterations in the number and function of immune cells, including T cells and monocytes, have been observed in MDD patients (13). Increased proportions of T helper 1 (Th1) and Th17 cells, coupled with impaired regulatory T cell (Treg) function, may exacerbate inflammatory responses (13). Microglia, the resident immune cells of the brain, play a pivotal role in the pathogenesis of MDD (14, 15). Under inflammatory stimuli, microglia become activated, releasing pro-inflammatory cytokines that further amplify neuroinflammation. Additionally, microglia participate in synaptic pruning; their overactivation may lead to synaptic dysfunction, adversely affecting mood and cognitive function (14). Considering the involvement of immunological dysregulation in the etiology of MDD, immunomodulatory treatments have surfaced as a viable avenue for investigation. Anti-inflammatory medications, including TNF-α inhibitors and IL-6 receptor antagonists, may reduce inflammation and alleviate depressed symptoms (8). Additional exploration of the interaction between the immune system and MDD will enhance the knowledge of its pathophysiology and aid in the creation of more effective treatment strategies.

Multi-omics integration transcends the limitations of single-layer analyses. It enables cross-verification of research findings, reveals cell type-specific dynamics obscured in bulk data, and uncovers functional interactions across molecular layers. This study aims to systematically decipher the molecular regulatory network of MDD by integrating transcriptomics and single-cell sequencing data. This approach will not only provide novel insights into the pathological mechanisms of MDD but also identify precise therapeutic targets for clinical diagnosis and targeted interventions.

## 2 Materials and methods

### 2.1 Data sources and ethical statements

**Figure 1:**
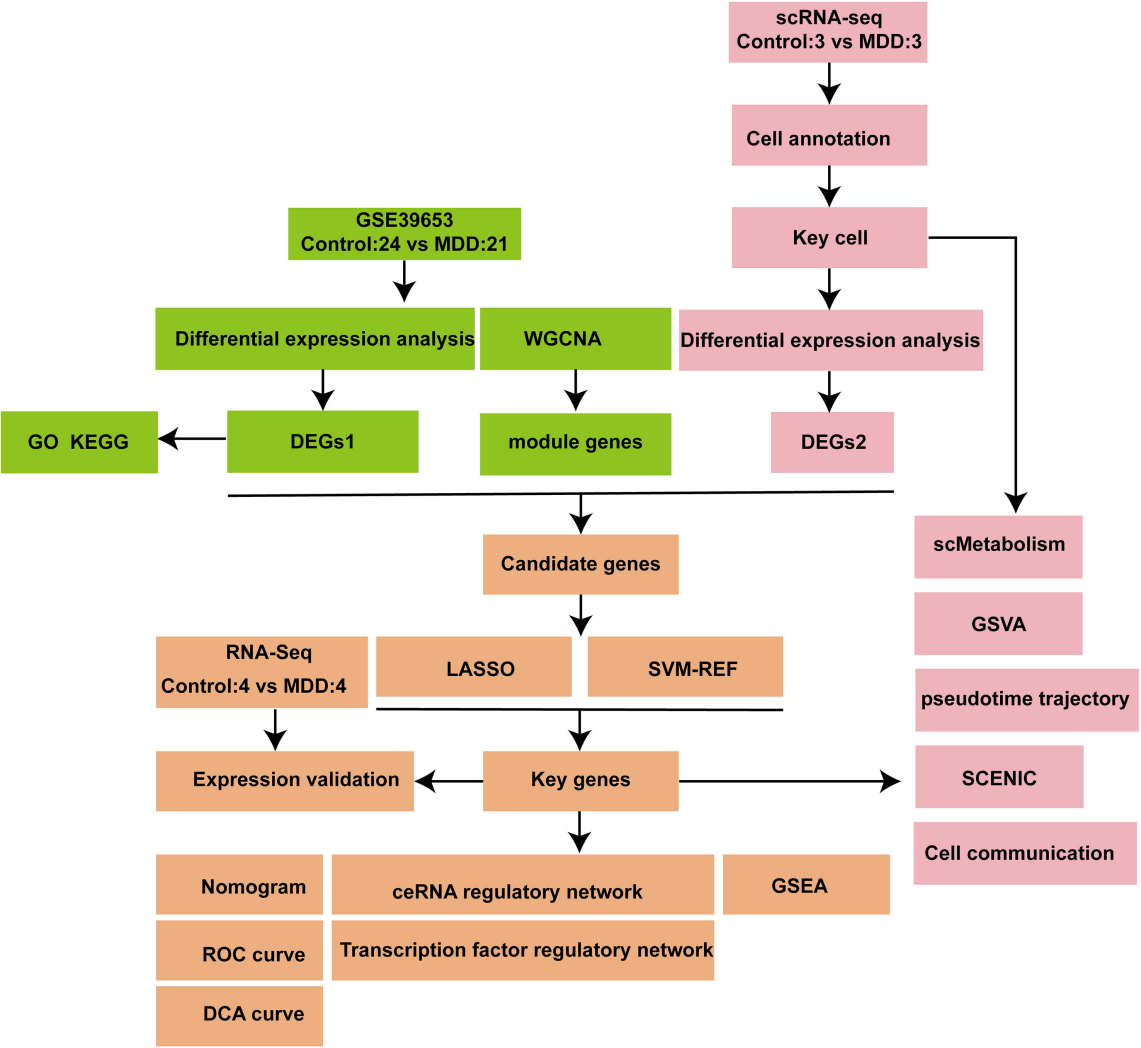
Flow chart.

This study integrated original clinical specimens with public database resources. Peripheral blood samples were collected from 4 patients with MDD and 4 healthy controls for transcriptome sequencing (RNA-seq), constituting Dataset1. Concurrently, single-cell RNA sequencing (scRNA-seq) was performed on samples from 3 MDD patients and 3 control. All clinical procedures were approved by the Ethics Committee of Yan’an Hospital affiliated to Kunming Medical University (No. 2025-055-01), and written informed consent was obtained from all participants. To enhance statistical power, this study downloaded the GSE39653 dataset from the Gene Expression Omnibus (GEO) database. This dataset, based on the GPL10558 microarray platform, comprises peripheral blood mononuclear cells (PBMCs) from a total of fifty-three samples. For subsequent analysis, all healthy control samples (n = 24) and MDD patient samples (n = 21) were selected and included (16). As this study focuses exclusively on depression, the 8 patients with bipolar disorder were excluded. The entire experimental process is shown in **Figure 1**.

### 2.2 Transcriptomic differential expression and functional annotation

Transcriptomic profiles from the GSE39653 dataset were analyzed to identify differentially expressed genes (DEGs1) between patients with MDD (n = 21) and healthy controls (n = 24) using the limma package (version 3.54.1). Genes meeting significance thresholds (|log_2_ fold-change| ≥ 0.1 and p-value < 0.05) were visualized through hierarchical clustering heatmaps (heatmap package v1.0.12) and expression distribution plots (ggplot2 v3.3.6) (17). Gene Ontology (GO) and Kyoto Encyclopedia of Genes and Genomes (KEGG) pathway enrichment analyses were subsequently performed on the DEGs1 using clusterProfiler (v4.4.4). Significantly enriched terms were identified with a false discovery rate (FDR) threshold of < 0.05. Results were visualized using ggplot2-generated (v3.3.6) dot plots and bar plots, with terms ordered by gene ratio and enrichment significance (18).

### 2.3 Identification of MDD-associated modules via weighted gene co-expression network analysis

Based on the GSE39653 dataset, a weighted gene co-expression network was constructed using the WGCNA package. First, cluster analysis was performed on all samples with results visualized, enabling the identification and removal of outlier samples. To build a co-expression network conforming to scale-free topology, we screened for the optimal soft thresholding power. Specifically, a threshold was selected where the scale-free topology fit index (R²) reached approximately 0.85, maximizing adherence to scale-free network distribution properties. Subsequently, using the determined soft threshold, the adjacency matrix between genes was calculated and further transformed into a topological overlap matrix (TOM). Gene dissimilarity was computed based on TOM, followed by hierarchical clustering to generate a gene dendrogram. For module identification, the dynamic hybrid tree cutting algorithm was applied to partition the dendrogram, with a minimum module size set to 100 genes. To identify key gene modules associated with MDD, MDD was treated as a phenotypic trait. The correlation between module eigengenes and this trait was calculated, and modules showing statistically significant correlations with MDD were designated as key modules. Genes contained within these key modules were defined as module member genes (19).

### 2.4 Single-cell transcriptomic data processing

Rigorous quality control was applied to single-cell data, excluding cells with > 10% mitochondrial gene content, total UMIs ≥ 30,000, or detected genes < 200 or > 6,000. Following integration of expression matrices from six samples using Seurat (v4.3.0), log-normalization and variance stabilization were performed. The top 2,000 highly variable genes (HVGs) were selected for downstream analyses. Dimensionality reduction was conducted via principal component analysis (top 30 PCs retained), followed by unsupervised clustering using k-nearest neighbor graphs (k = 20) and the Louvain modularity optimization algorithm (resolution = 0.8). Following dimensionality reduction and clustering via Uniform Manifold Approximation and Projection (UMAP), cell clusters were manually annotated to specific cell types by referencing canonical marker genes described in existing literature. The proportional differences of these annotated subpopulations between the MDD and control groups were then quantified (20, 21).

To comprehensively delineate cellular dynamics and intercellular communication patterns, CellChat (v1.6.0) was applied, a computational tool designed for the systematic inference and analysis of ligand-receptor-mediated signaling networks. By integrating curated ligand-receptor interaction databases from CellChatDB, communication probabilities among distinct cell types were identified and quantified, thereby revealing context-specific signaling pathways and functional networks (22). Concurrently, Monocle (v2.26.0) was utilized to construct pseudotemporal developmental trajectories of key subpopulations, simulating cellular state transitions.

### 2.5 Key cellular functions and gene screening

Within specific cell subpopulations, KEGG pathways showing significant enrichment (p<0.05) were identified through Gene Set Variation Analysis (GSVA). Metabolic pathway activities were assessed using scMetabolism (v0.2.1) (23), while transcription factor regulatory networks were inferred via the SCENIC method (24). Subsequently, cell type-specific differentially expressed genes (DEGs) were identified using the following criteria: expression in ≥ 10% of cells and |log_2_FC| > 0.25, defining the DEGs2 gene set.

### 2.6 Identification of key genes and diagnostic model construction

Candidate genes were identified through the intersection of DEGs1, modules genes, and DEGs2. To elucidate functional interactions among these candidate genes, a protein-protein interaction (PPI) network was constructed using the STRING database, employing a confidence score threshold of > 0.7 to include only high-confidence interactions. For further refinement of key genetic determinants, two distinct machine learning-based feature selection methods were applied: least absolute shrinkage and selection operator (LASSO) regression, implemented via the R package glmnet (v4.1.7), and support vector machine-recursive feature elimination (SVM-RFE) using the e1071 package (v1.7.13). The intersection of genes selected by both LASSO and SVM-RFE yielded a robust set of five key genes. Differential expression of these genes was statistically validated (p < 0.05) using independent t-tests in both the original Dataset1 and GSE39653 cohort.

### 2.7 Diagnostic model construction and validation

A logistic regression diagnostic model was constructed using the key genes, with variable contributions visualized via a nomogram. The performance of the diagnostic model was rigorously evaluated using multiple complementary approaches. Calibration curves were plotted to assess the agreement between predicted probabilities and actual outcomes, thereby indicating the accuracy of the model. Receiver operating characteristic (ROC) curves were employed to evaluate the discriminatory power of the model, with the area under the curve (AUC) serving as a metric of classification efficacy. Furthermore, decision curve analysis (DCA) was conducted to quantify the net clinical benefit across different threshold probabilities and to determine the practical utility of the model for clinical decision-making.

### 2.8 Regulatory mechanisms and functional validation of key genes

To elucidate the biological significance of key genes, Gene Set Enrichment Analysis (GSEA) was performed on the GSE39653 dataset. Genes were ranked by their correlation with key genes, and the gseKEGG function identified co-regulated pathways (p < 0.05). For regulatory network analysis, miRNAs (threshold: TDMD score ≥ 1, ≥ 5 CLIP-seq evidence counts) and their targeted lncRNAs (≥ 20 CLIP-seq supports) were predicted via StarBase to construct a ceRNA interaction network. Potential compounds regulating key gene expression were screened from the Comparative Toxicogenomics Database (CTD), requiring ≥ 2 literature supports and consistent regulation direction. Finally, transcriptional regulatory data for NK cell activity factors were integrated to establish a comprehensive transcription factor-protein interaction network using STRING.

### 2.9 Isolation and flow cytometric analysis of natural killer cells from human peripheral blood

This study employed flow cytometry (model: Attune NxT, Thermo Fisher Scientific Inc.) to determine the frequency of CD3^-^(Cat. 2488551, Thermo Fisher Scientific Inc.) CD56^+^(Cat. 2841493, Thermo Fisher Scientific Inc.) or CD3^-^CD16^+^(Cat.360705, BioLegend, Inc.) natural killer (NK) cells in human peripheral blood samples. Peripheral blood mononuclear cells (PBMCs) were isolated from 10 human peripheral blood samples using density gradient centrifugation. Briefly, anticoagulated whole blood was centrifuged, and the pelleted cells were diluted with an equal volume of PBS. This mixture was then carefully layered onto lymphocyte separation medium and centrifuged. The PBMC layer was collected, subjected to red blood cell lysis, and washed. For immunophenotyping, cells were stained with fluorescently conjugated antibodies against CD3 (FITC) and CD56/16 (PE), with appropriate single-stained and unstained controls included. Antibody incubation was performed at room temperature in the dark, followed by washing steps. Samples were resuspended in staining buffer and analyzed on a Thermo Fisher Attune NXT flow cytometer. Lymphocytes were gated based on forward and side scatter properties, doublets were excluded, and the percentage of CD3-negative, CD56-positive cells or CD16-positive cells within the lymphocyte population was determined.

## 3 Results

### 3.1 Differential expression analysis of the transcriptome

**Figure 2:**
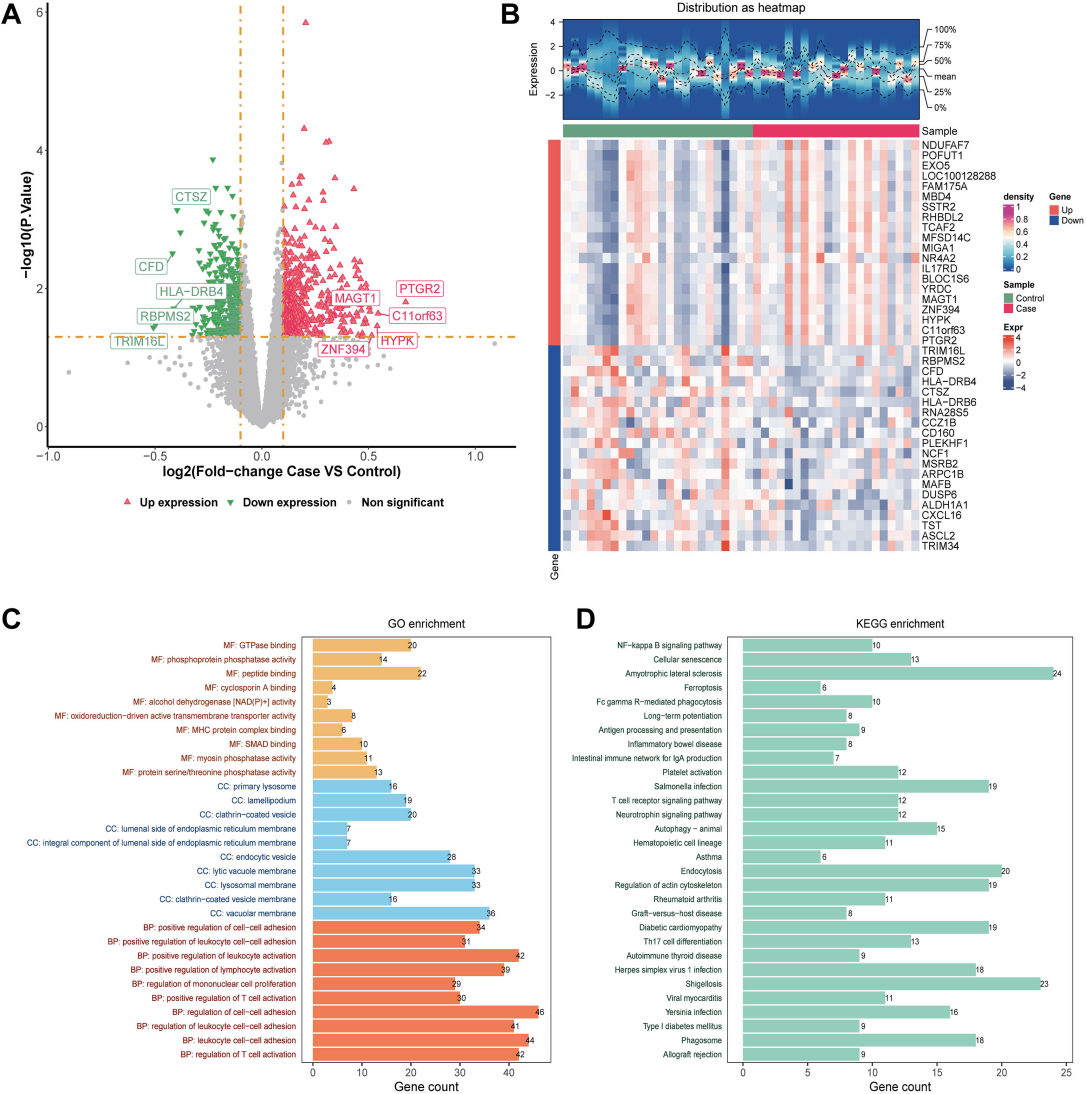
Identification and enrichment analysis of DEGs1. **(A)** Volcano plot displaying the DEGs1. |log_2_ fold-change| ≥ 0.1 and p-value < 0.05. The genes that were up- or down-regulated in the TOP5 were annotated; **(B)** Heatmap of the expression patterns of the top 20 DEGs1 across samples. Rows represent genes and columns represent individual samples. The color key indicates normalized expression values, with red denoting high expression and blue denoting low expression; **(C)** GO enrichment analysis of DEGs1, showing the top 30 significantly enriched terms in GO item. **(D)** KEGG pathway enrichment analysis of DEGs1, showing the top 30 significantly enriched pathways.

Differential expression analysis identified 803 DEGs1 within the GSE39653 dataset, comprising 454 upregulated and 349 downregulated genes (**Figure 2A**). The top 20 up- and down-regulated DEGs1 are shown in the heatmap (**Figure 2B**). Subsequent GO enrichment analysis of these DEGs revealed significant enrichment in key biological processes (BP), cellular components (CC), and molecular functions (MF), including regulation of cell-cell adhesion (BP), vacuolar membrane (CC), and peptide binding. KEGG pathway enrichment analysis further indicated that the DEGs1 were predominantly enriched in the Amyotrophic lateral sclerosis, Shigellosis, and Endocytosis signaling pathways (**Figures 2C, D**).

### 3.2 Identification of 2,080 module genes via WGCNA in the GSE39653 dataset

**Figure 3:**
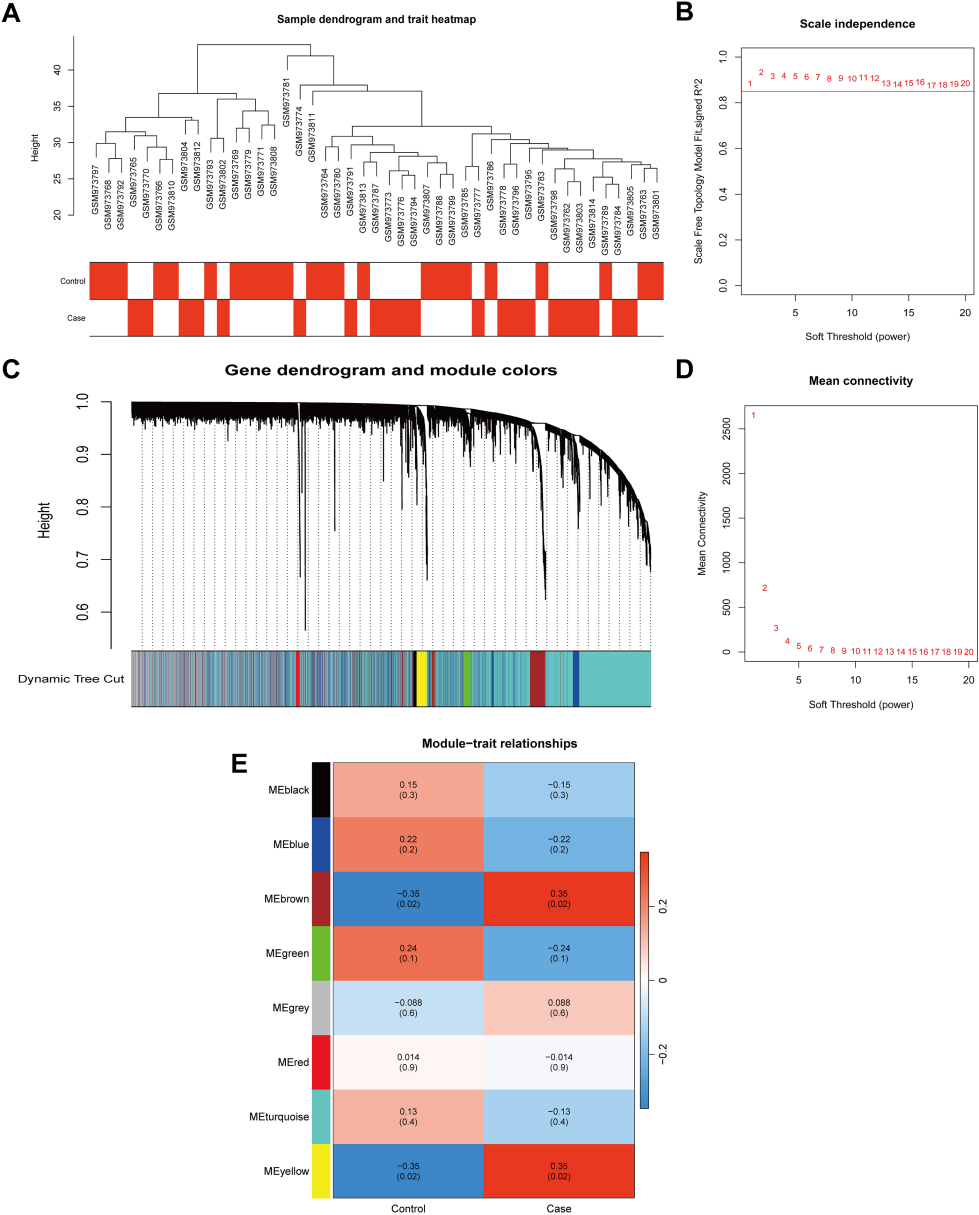
Identification of co-expression gene modules using WGCNA. **(A)** Sample clustering and trait heatmap; **(B, D)** Analysis of network topology for various soft-thresholding powers. The selected power (β = 6) was the lowest value at which the scale-free topology fit index reached (red line), balancing scale-free topology and network connectivity; **(C)** Cluster dendrogram of genes; **(E)** The resulting eight co-expression modules.

Sample clustering analysis of the GSE39653 dataset revealed no outliers (**Figure 3A**). To establish a biologically relevant scale-free co-expression network, a soft-thresholding power (β) of 6 was selected based on a scale independence criterion exceeding 0.85 (**Figures 3B-D**). Eight distinct co-expression modules were subsequently identified. Among these, the MEbrown (r = 0.35) and MEyellow (r = 0.35) modules demonstrated significant correlation with the MDD traits, collectively containing 2,080 module genes (**Figure 3E**).

### 3.3 NK cells are identified as key cellular players in MDD

**Figure 4:**
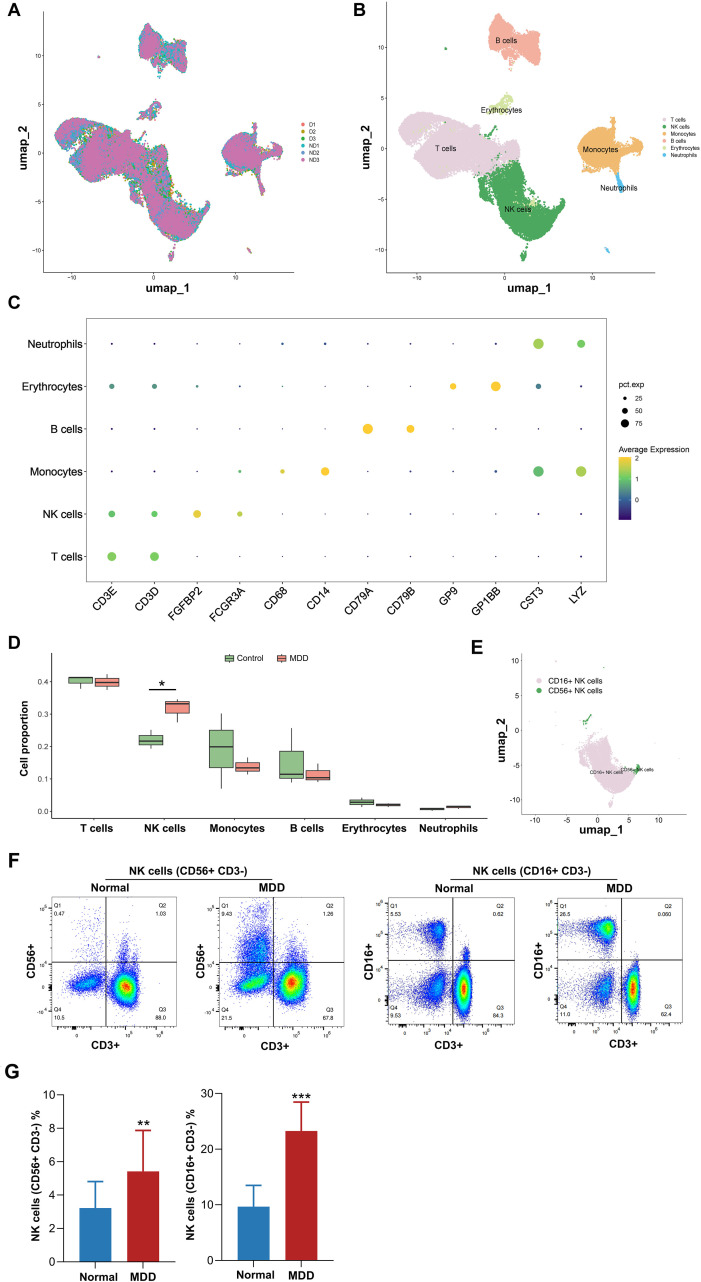
Sc-RNA sequencing analysis reveals cell type composition and key alterations in MDD. **(A, B)** UMAP visualization of single-cell clusters **(A)** before and **(B)** after cell type annotation; **(C)** Dot plot of canonical marker gene expression used for cell type identification. Dot size represents the percentage of cells within a cluster expressing the indicated gene, and color intensity reflects the average expression level; **(D)** Bar graph comparing the proportional abundance of a key cell subtype between Control and MDD groups. Statistical significance (^*^p < 0.05). **(E)** Based on the expression of CD16 (FCGR3A) and CD56 (NCAM1), NK cells were classified into two subsets, CD16^+^NK cells and CD56^+^NK cells. **(F)** The proportions of CD3-CD56+ and CD3-CD16+ cells were analyzed by flow cytometry and visualized through scatter plots. **(G)** The proportions of CD3-CD56+ cells and CD3-CD16+ cells in the whole blood of normal individuals and MDD patients were presented in a bar chart. Normal group vs. MDD group, ^**^P<0.01, ^**^P<0.001.

Quality control metrics for the scRNA-seq data are presented in **Supplementary Figure 1**. The analysis incorporated 56,122 high-quality cells and 27,882 genes. Principal component analysis (PCA) identified 30 principal components (PCs) significant for downstream analysis. Cells were subsequently clustered into 4 distinct populations using the UMAP algorithm (**Figure 4A**). Based on established marker gene expression patterns, these clusters were annotated as six major immune cell types: T cells, NK cells, monocytes, B cells, erythrocytes, and neutrophils (**Figure 4B**). Differentially expressed marker genes across these cell types are shown in **Figure 4C**. Comparative analysis of cell type proportions between the Control and MDD groups revealed a significantly elevated proportion of NK cells in the MDD group. Therefore, NK cells are implicated as key cellular players in the pathophysiology of MDD (**Figure 4D**). Based on the expression of CD16 (FCGR3A) and CD56 (NCAM1), NK cells were classified into two subsets, CD16^+^NK cells and CD56^+^NK cells (**Figure 4E**). Flow cytometry was performed on peripheral blood samples from the normal and MDD groups (**Figure 4G**). The proportion of NK cells (CD16^+^ or CD56^+^) were significantly increased in the MDD group, compared with the control group (p<0.001) (**Figure 4F**). These results indicate that NK-cell expansion represents the most prominent immune alteration in MDD and provides cellular-level validation for the key genes and mechanisms identified in the study.

### 3.4 Functional annotation of NK cells

**Figure 5:**
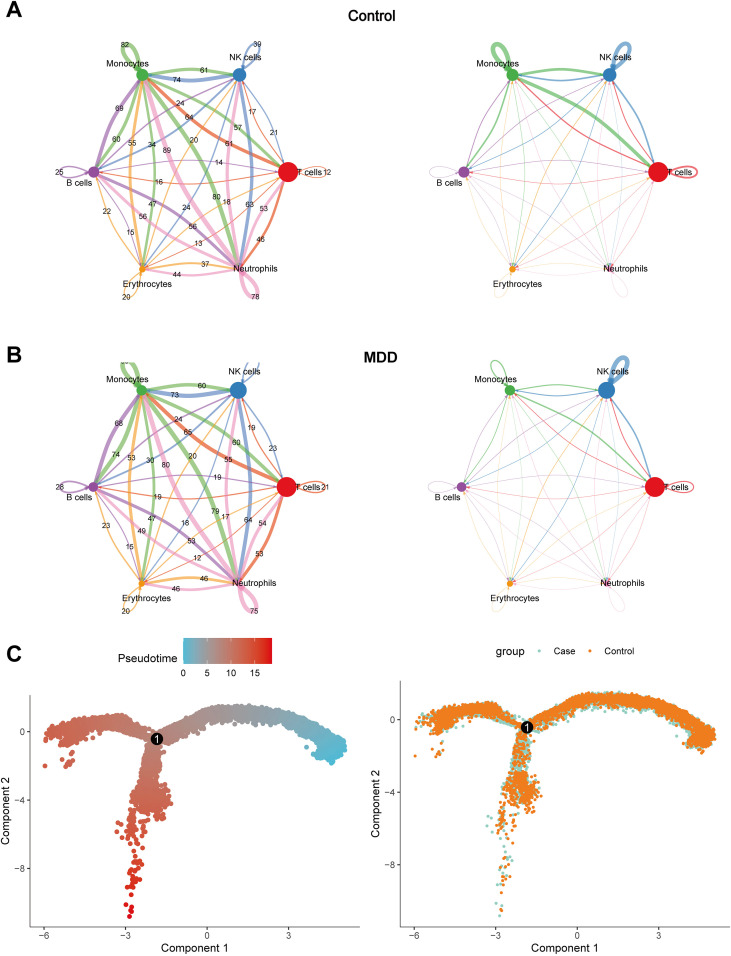
Analysis of intercellular communication and developmental trajectories reveals altered signaling and cell states in MDD. **(A, B)** Inference of cell-cell communication networks; **(C)** Reconstruction of cellular differentiation trajectories.

Immunocyte interaction network analysis of the Control group revealed T cells acting as central coordinators, transmitting high-frequency signals to monocytes and significantly activating neutrophils, while neutrophils formed the strongest effector axis with B cells. In contrast, the MDD group exhibited significant immune network remodeling, characterized by T cell functional inactivation with a sharp reduction in interactions towards NK cells; concurrently, interactions between neutrophils and B cells were diminished. NK cell autocrine signaling was enhanced (**Figures 5A, B**). Pseudotime trajectory analysis performed exclusively on NK cells revealed no discernible segregation pattern between MDD and Control groups along the reconstructed developmental continuum (**Figure 5C**).

**Figure 6:**
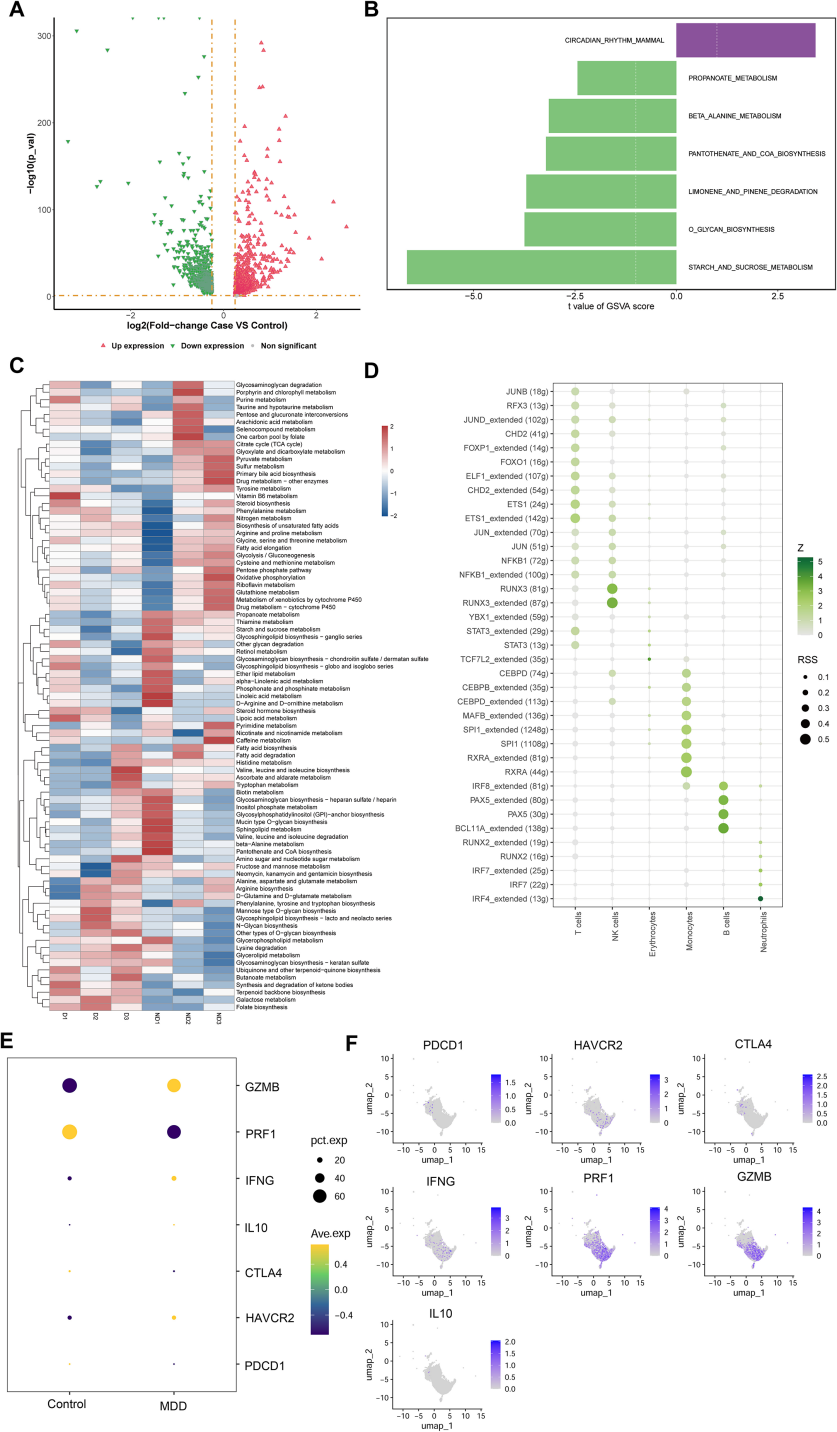
Functional annotation of NK cells. **(A)** Volcano plot of DEGs2 in NK cells between Control and MDD; **(B)** GSVA enrichment scores of hallmark signaling pathways in NK cells; **(C)** scMetabolism analysis quantifying the activity of metabolic pathways in NK cells; **(D)** SCENIC analysis revealing transcription factor (TF) regulation activity in NK cells. **(E)** The dot plot shows the expression patterns of key cytotoxic and immunoregulatory genes in immune cells from healthy controls and patients with major depressive disorder (MDD). Dot size corresponds to the percentage of cells expressing each gene (pct.exp), while color intensity represents the average scaled expression (Ave.exp). **(F)** The UMAP feature map shows the spatial distribution and expression intensity of representative immune-related genes PDCD1, HVCR2, CTLA4, IFNG, PRF1, GZMB, and IL10 in single-cell clusters.

Transcriptomic profiling of NK cells initially identified 1,539 DEGs2. Subsequent filtration based on statistical significance (p < 0.05) yielded 1,523 robust DEGs2, comprising 698 upregulated genes and 825 downregulated transcripts (**Figure 6A**). GSVA of these DEGs2 demonstrated significant enrichment (p < 0.05) for key biological processes including Glycosaminoglycan biosynthesis - keratan sulfate, Terpenoid backbone biosynthesis, Aminoacyl-tRNA biosynthesis, and RNA degradation (**Figure 6B**). Furthermore, GSVA quantified functional pathway activity disparities between cohorts, identifying seven pathways exhibiting statistically significant differential activity. Notably, the circadian rhythm mammal pathway displayed elevated activity in MDD samples, whereas diminished activity characterized MDD samples in starch and sucrose metabolism. Metabolic pathway profiling identified constitutively high-activity pathways in NK cells including Glycosphingolipid biosynthesis - lacto and neolacto series, Folate biosynthesis, and Alanine, aspartate and glutamate metabolism. Comparative analysis revealed significant inter-group differences (p < 0.05) in specific metabolic pathways: Ubiquinone and other terpenoid-quinone biosynthesis, Butanoate metabolism, Synthesis and degradation of ketone bodies, Terpenoid backbone biosynthesis, Galactose metabolism, and Folate biosynthesis exhibited altered activity in MDD specimens (**Figure 6C**). Transcription factor activity assessment identified RUNX3, JUND, NFKB1, JUN, RELB, JUNB, and FOS as exhibiting predominant activity within the NK cell compartment (**Figure 6D**).

To characterize the immunoregulatory status of NK cells, we examined the expression of classical activation, exhaustion, and cytotoxicity-related genes at the single-cell level. PDCD1 (PD-1), HAVCR2 (TIM-3), CTLA4, and IL10 displayed minimal expression in NK cells across both groups, with no substantial Case-Control differences. IFNG showed generally low expression, with a modest increase in the Case group. In contrast, cytotoxic effector molecules PRF1 and GZMB were robustly expressed in NK cells in both conditions, consistent with preserved NK-cell cytotoxicity (**Figure 6E**). As shown in the UMAP feature plots, PDCD1, HAVCR2, CTLA4, and IL10 are expressed at very low levels and are sparsely distributed across NK-cell clusters, indicating that NK-cell exhaustion or inhibitory programs are not prominently activated. IFNG shows weak but detectable expression in a small NK-cell subset. In contrast, PRF1 and GZMB display widespread and high expression within the major NK-cell cluster, confirming that NK cells predominantly maintain a cytotoxic phenotype. These findings are consistent with our dot-plot analysis and further support the preserved cytotoxic function of NK cells in the Case group (**Figure 6F**).

### 3.5 Identification of 5 key genes for MDD

**Figure 7:**
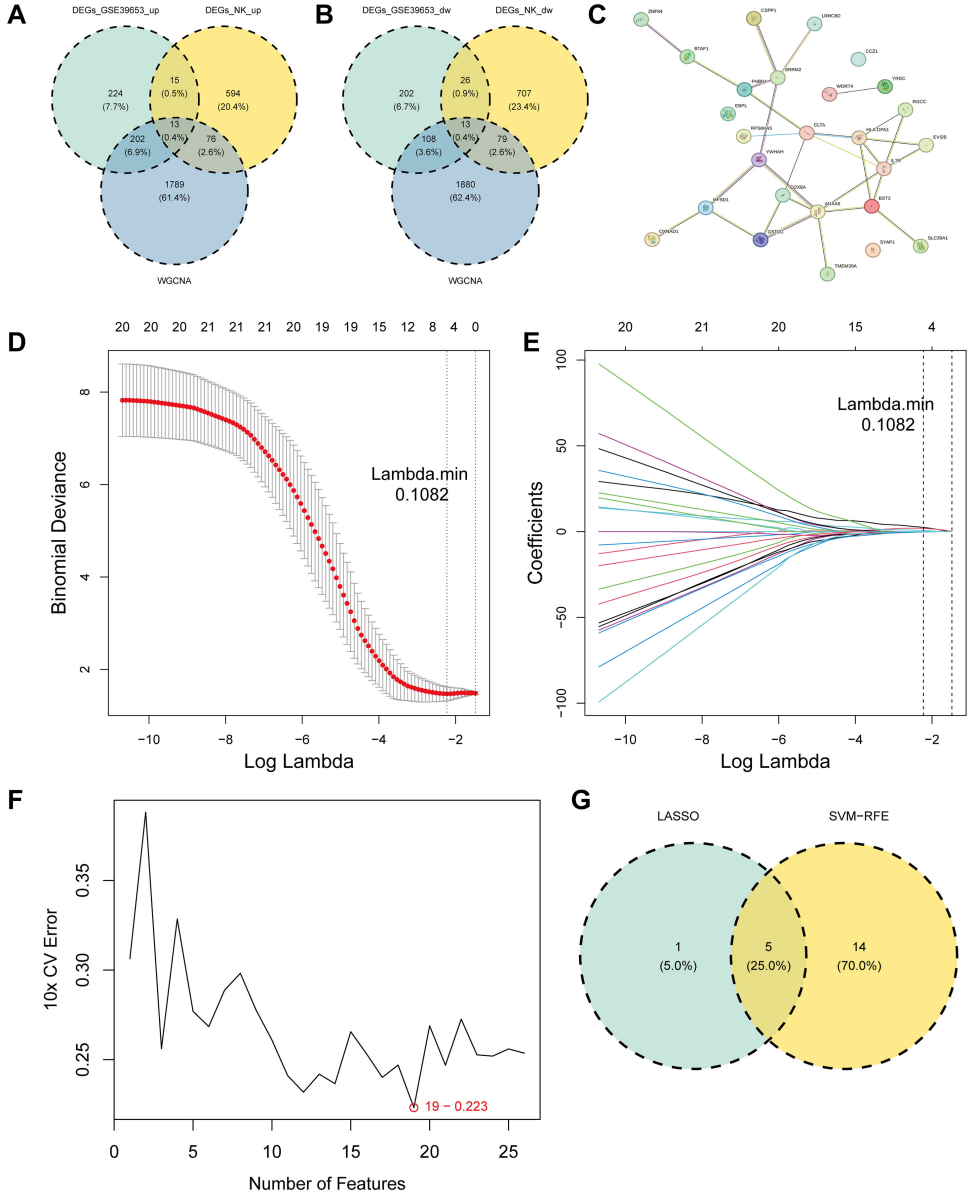
Identification of key genes through integrative bioinformatics and machine learning approaches. **(A, B)** Venn diagrams illustrating the intersection of candidate genes derived from DEGs1, DEGs2 and modules keys; **(C)** PPI network of candidate genes constructed using the STRING database (interaction score > 0.7); **(D, E)** Feature selection using the LASSO regression model; **(F)** Feature selection using SVM-RFE algorithm; **(G)** Venn diagram identifying the key genes by intersecting the optimal feature sets derived from both LASSO and SVM-RFE algorithms.

By intersecting DEGs1, module genes, and DEGs2, we identified 13 upregulated and 13 downregulated genes (**Figures 7A, B**). A protein-protein interaction (PPI) network for these 26 intersecting genes was constructed using the STRING database, comprising 26 nodes and 30 interaction edges (**Figure 7C**). LASSO regression analysis on the 26 genes identified 6 feature genes, centrosome and spindle pole associated protein 1(CSPP1), zinc finger protein 84 (ZNF84), regulator of cell cycle (RGCC), major histocompatibility complex, class II, DP alpha 1 (HLA-DPA1), CCZ1 vacuolar protein trafficking and biogenesis associated (CCZ1), leucine rich repeat containing 8 VRAC subunit D (LRRC8D) at lambda.min = 0.1082 (**Figures 7D, E**). SVM-RFE analysis on the same set selected an optimal model containing 19 feature genes (**Figure 7F**). Integration of LASSO and SVM-RFE results via Venn diagram identified 5 key genes: CSPP1, ZNF84, HLA-DPA1, CCZ1, and LRRC8D (**Figure 7G**).

### 3.6 Validated expression patterns and diagnostic model performance

**Figure 8:**
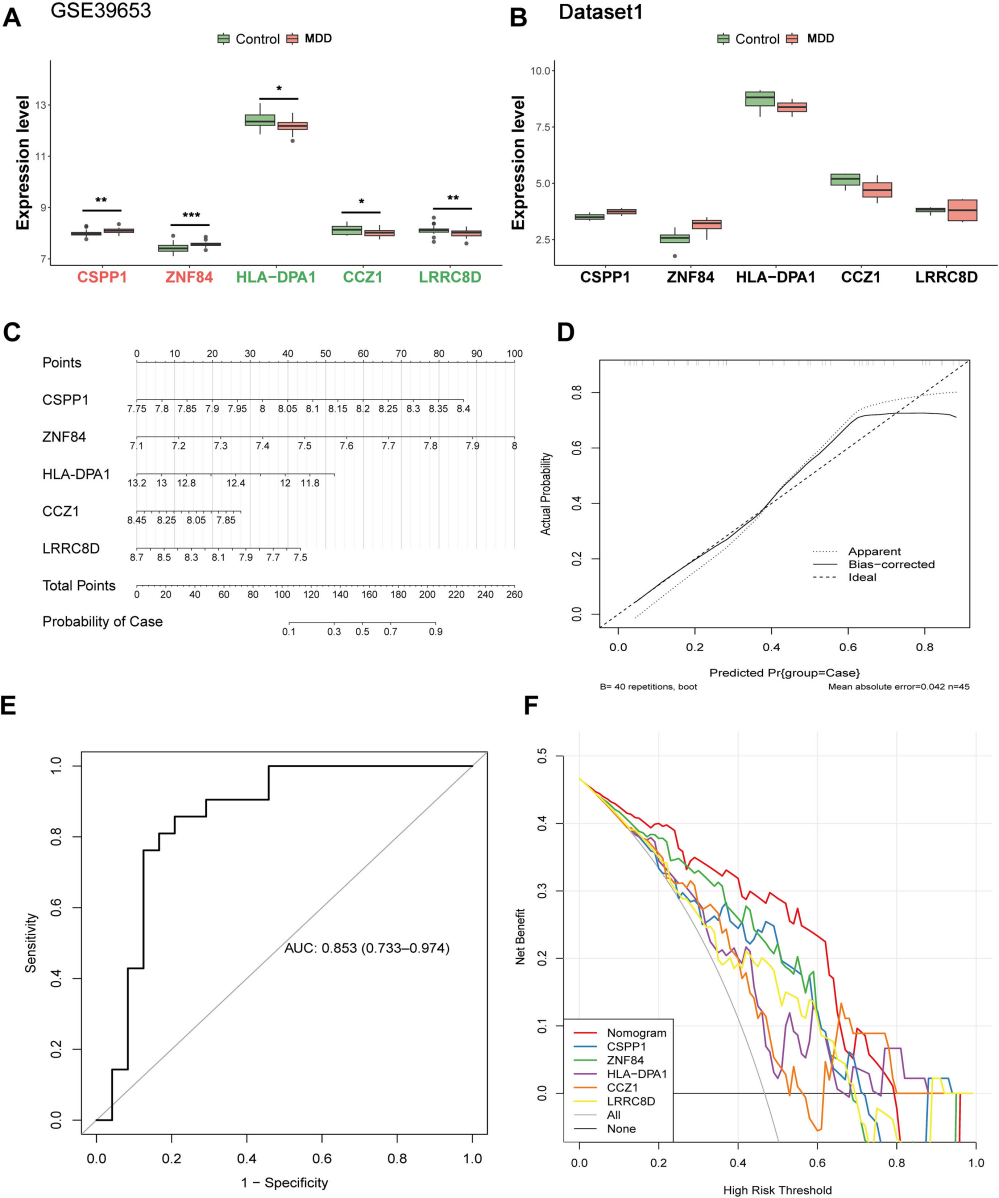
Validation of key gene expression and evaluation of the diagnostic model performance. **(A, B)** Validation of the expression patterns of the 5 key genes in two independent datasets (GSE39653 dataset and Dataset1). Statistical significance between control and MDD groups was determined by independent sample t-test (*p < 0.05, **p < 0.01, ***p < 0.001); **(C)** Nomogram constructed based on the expression levels of the key genes for visual prediction of disease probability. Each gene is assigned a points scale; the total points calculated from all genes are directly mapped to the risk of disease occurrence; **(D)** Calibration curve evaluating the agreement between the nomogram-predicted probability and the actual observed outcome. **(E)** ROC curves demonstrating the diagnostic performance of the key gene-based model; **(F)** DCA evaluating the clinical utility of the diagnostic model.

Validation of the expression patterns of these five key genes was performed in both the GSE39653 dataset and the Dataset1. The results demonstrated that in the GSE39653 dataset, CSPP1 (p < 0.01) and ZNF84 (p < 0.001) were significantly upregulated in MDD samples, while HLA-DPA1 (p < 0.05), CCZ1 (p < 0.05), and LRRC8D (p < 0.01) were significantly downregulated. However, in the Dataset1, none of the five key genes showed significant differences between control and MDD groups. Nevertheless, the expression trends of CSPP1, ZNF84, HLA-DPA1, and CCZ1 remained consistent with those observed in the GSE39653 dataset (**Figures 8A, B**). A diagnostic logistic regression model was constructed based on these five key genes and visualized using a nomogram (**Figure 8C**). Model performance evaluation demonstrated: a calibration curve slope approaching the ideal value of 1; an area under the ROC curve (AUC) of 0.853; and decision curve analysis (DCA) indicating that the model provided higher net benefit than single-gene predictions (**Figures 8D-F**).

### 3.7 Functional enrichment analysis of key genes

**Figure 9:**
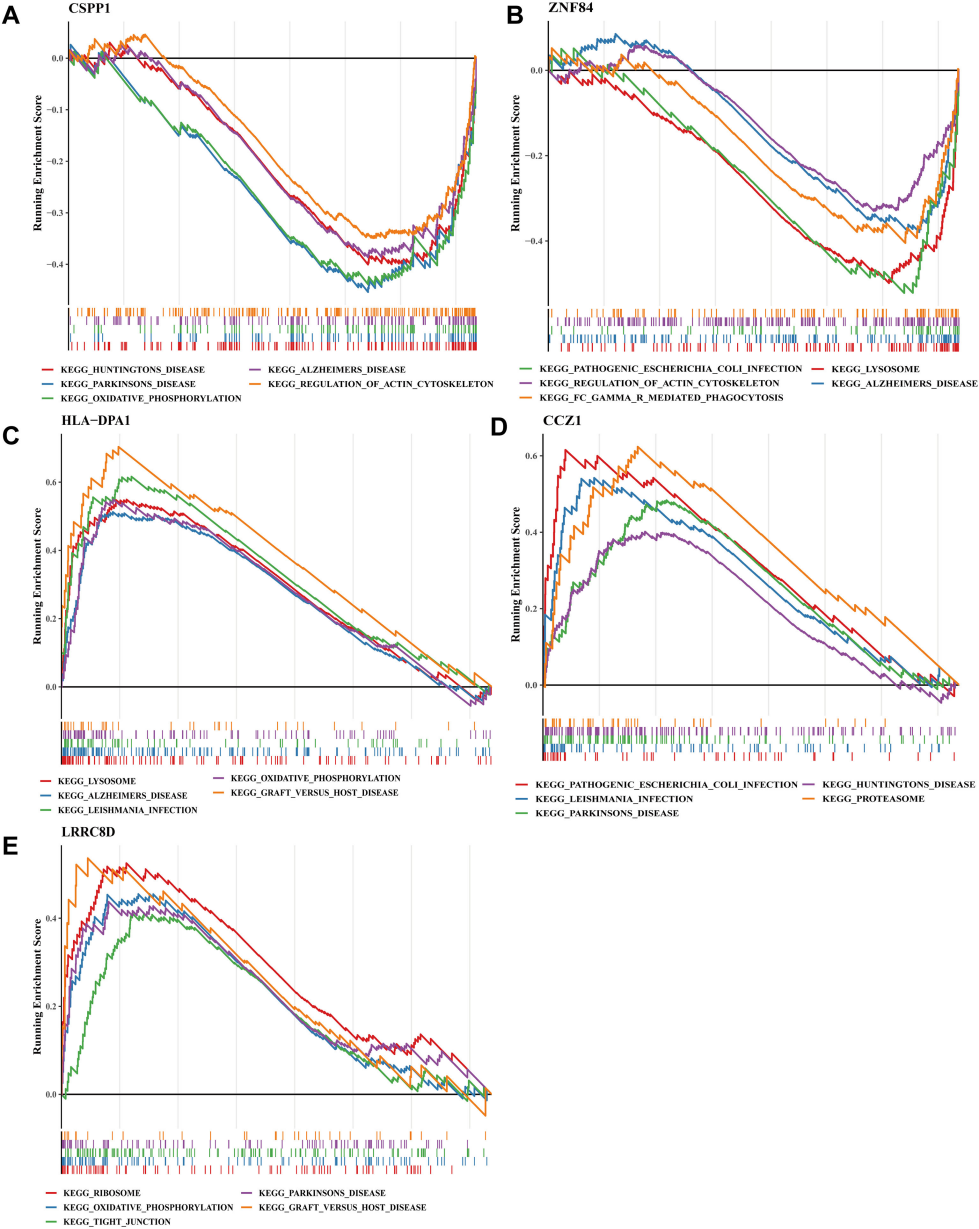
GSEA analysis of key genes. **(A-E)** GSVA analysis of CSPP1, ZNF84, HLA-DPA1, CCZ1, and LRRC8D.

The GSEA was performed to characterize the functional pathways associated with the expression patterns of the five key genes in MDD. For CSPP1, the negatively enriched KEGG terms primarily involved mitochondrial and cytoskeletal processes, including oxidative phosphorylation and regulation of the actin cytoskeleton, alongside several canonical disease-named KEGG modules (e.g., Huntington’s disease, Parkinson’s disease, Alzheimer’s disease), which reflect shared pathway components rather than clinical disease associations. ZNF84 showed negative enrichment in lysosomal function, cytoskeletal regulation, and immune-related modules such as FC gamma R–mediated phagocytosis and pathogenic Escherichia coli infection. In contrast, HLA-DPA1 exhibited positive enrichment in immune and metabolic pathways, including lysosome, oxidative phosphorylation, Leishmania infection, and graft-versus-host disease. CCZ1 was positively associated with infection-related, neurodegeneration-related, and proteostasis pathways, including proteasome and multiple KEGG canonical modules. LRRC8D showed enrichment in translational and barrier-related pathways such as ribosome, tight junction, and oxidative phosphorylation. Collectively, these enrichment patterns highlight dysregulated metabolic, immune, and structural pathways linked to the expression of these genes in MDD (**Figures 9A-E**).

### 3.8 Construction of key gene regulatory networks and chemical–gene interaction analysis

**Figure 10:**
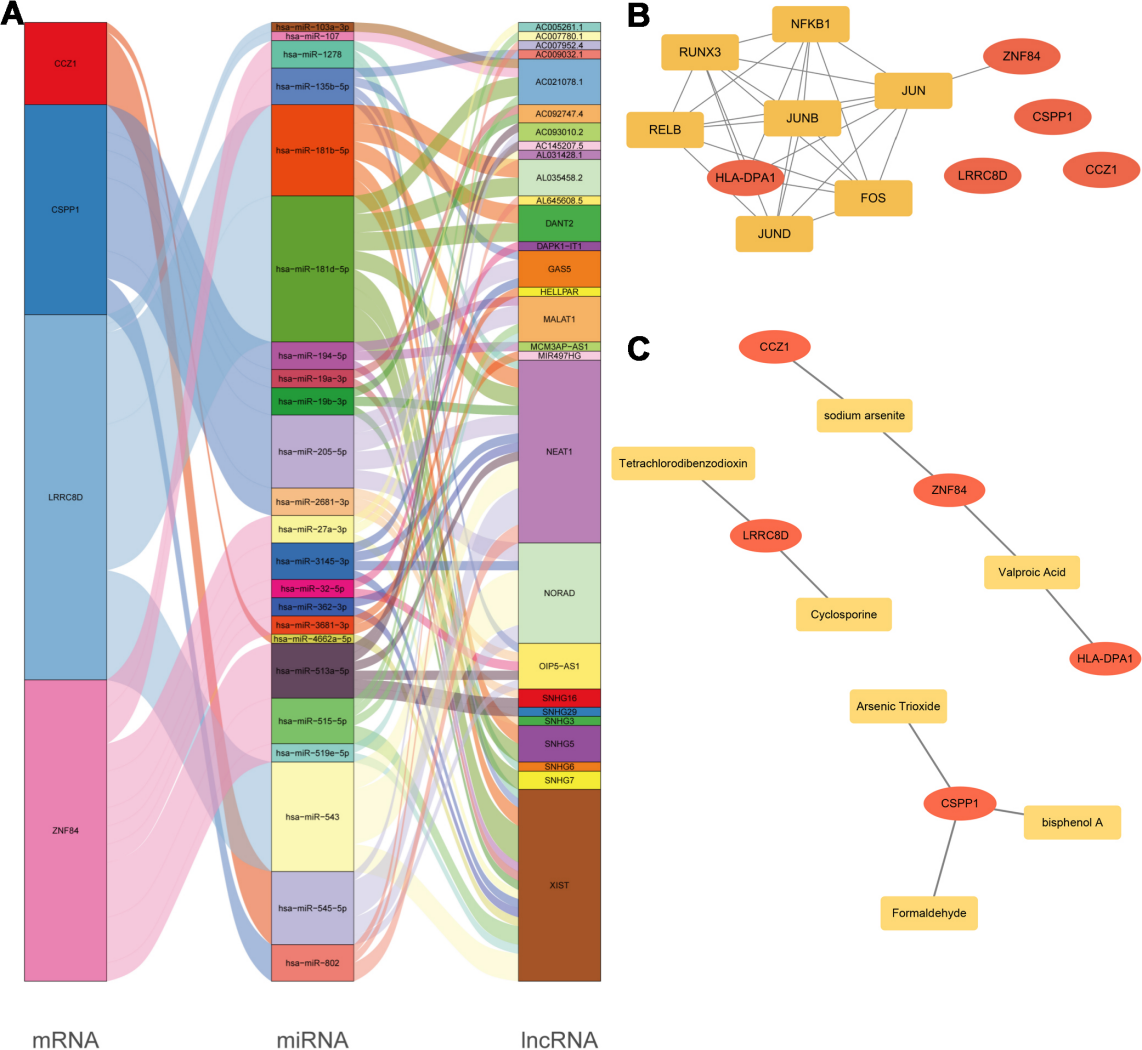
Integrated regulatory networks of key genes and related compound screening in MDD. **(A)** CeRNA network showing interactions among key mRNAs (CCZ1, CSPP1, LRRC8D, and ZNF84), 23 miRNAs, and 28 lncRNAs. Notably, lncRNA SNHG16 may act as a sponge for miR-513a-5p to regulate ZNF84 expression. **(B)** Transcription factor regulatory network indicating that ZNF84 is mainly regulated by JUN, while HLA-DPA1 is co-regulated by JUN, JUND, RUNX3, and NFKB1. **(C)** Gene–compound network based on CTD analysis. CSPP1 is regulated with arsenic trioxide, bisphenol A, and formaldehyde. ZNF84 is regulated with sodium arsenite and valproic acid. HLA-DPA1 is regulated with valproic acid. CCZ1 is linked to sodium arsenite, whereas LRRC8D is regulated by cyclosporine and tetrachlorodibenzodioxin.

The ceRNA regulatory network analysis identified potential regulatory interactions between important genes and 28 lncRNAs/23 miRNAs (**Figure 10A**). lncRNA SNHG16 may act as a molecular sponge for miR-513a-5p, hence regulating ZNF84 expression levels and affecting the course of MDD. Key gene regulatory networks including transcription factors (TFs) revealed that ZNF84 is controlled by JUN, whereas HLA-DPA1 is co-regulated by several TFs, including JUN, JUND, RUNX3, and NFKB1 (**Figure 10B**). Using the CTD database, we identified compounds regulating the five key genes based on two criteria: a reference count ≥2 and regulatory direction consistent with our hypothesis (downregulating CSPP1 or ZNF84, or upregulating HLA-DPA1, CCZ1, or LRRC8D). Seven compounds met these requirements and were incorporated into the gene–compound network (**Figure 10C**). CSPP1 was regulated with arsenic trioxide, bisphenol A, and formaldehyde. ZNF84 was regulated with sodium arsenite and valproic acid. HLA-DPA1 was regulated with valproic acid. CCZ1 was linked to sodium arsenite, whereas LRRC8D was regulated by cyclosporine and tetrachlorodibenzodioxin. These results outline a preliminary regulatory landscape between key genes and candidate compounds.

## 4 Discussion

In the GSE39653 dataset, we identified 803 DEGs1. Functional enrichment analysis revealed that DEGs1 were predominantly enriched in the regulation of cell adhesion, vacuolar membrane-related processes, and peptide binding. KEGG pathway enrichment analysis further demonstrated that DEGs1 were significantly enriched in pathways associated with amyotrophic lateral sclerosis (ALS), shigellosis, and endocytosis. Collectively, these findings suggest that the molecular pathology of MDD is closely linked to significant immune system activation and neuroinflammatory responses. At the functional level, the enrichment of DEGs1 in cell adhesion regulation, peptide binding (including antigen recognition via MHC molecules), and vacuolar membrane processes (involving intracellular degradation and autophagic homeostasis) implies crucial roles for immune cell interactions, antigen presentation, and the maintenance of intracellular homeostasis (25–27). At the pathway level, the significant enrichment in shigellosis (a pathway involving host immune responses to bacterial infection) and endocytosis (closely related to immune cell phagocytosis of pathogens and antigen presentation) further underscores the centrality of immune-inflammatory processes in MDD (28). Notably, the significant enrichment of DEGs1 in the ALS pathway revealed potential neuronal structural and functional abnormalities in MDD pathology, including impaired axonal transport, glutamate excitotoxicity, and neuroinflammation mediated by glial cells (8, 11). These results robustly support a strong association between MDD and immune system dysregulation.

Analysis of single-cell transcriptomic data identified six major immune cell types: T cells, NK cells, monocytes, B cells, erythrocytes, and neutrophils. Notably, the proportion of NK cells was significantly elevated in the MDD group. Cell-cell interaction analysis revealed that T cells exhibited the most extensive interaction network with other cell types. Compared to the control group, significant differences were observed in the interaction patterns of monocytes and NK cells with other cells in the MDD group. Given these proportional changes and interactional differences, this study focused on NK cells for in-depth investigation. NK cells, as critical effector cells of the innate immune system, recognize and lyse virus-infected or tumor cells, primarily through the release of cytotoxic granules, and secrete cytokines such as interferon-gamma (IFN-γ) and tumor necrosis factor-alpha (TNF-α) to modulate immune responses (8). Previous studies have shown a significant reduction in DNA methylation-based estimates of NK cell proportions in untreated MDD patients (29). Chronic stress has been shown to reduce NK cell numbers in the bone marrow and circulation of both mice and humans (30, 31). Compared to healthy controls, MDD patients exhibit a reduced proportion of the CD56+CD16− NK cell subset (the primary producer of IFN-γ) and lower serum IFN-γ levels (32–34), suggesting a potential association between NK cell dysfunction and inflammation-related depression development. Furthermore, acute psychological stress has been found to transiently increase NK cell counts (35). This bidirectional dynamic indicates that acute stress precipitating suicide may upregulate NK cells, while chronic stress downregulates them, potentially correlating with MDD severity. Additionally, the age of depression onset has been associated with reduced NK cell numbers and activity (36). Our findings demonstrate significant immune network remodeling in MDD patients, characterized by T-cell functional inactivation with markedly weakened interactions with NK cells, reduced interaction strength between neutrophils and B cells, and enhanced autocrine signaling in NK cells. These immune aberrations may trigger a cascade of pathological effects: T-cell suppression could impair pathogen clearance, exacerbating chronic inflammation and neural damage; diminished NK cell interactions may compromise immune surveillance, increasing susceptibility to infections and tumors while worsening inflammatory dysregulation; weakened neutrophil-B cell interactions might lead to insufficient immune responses, affecting antibody production and inflammation regulation; enhanced NK cell autocrine signaling could promote cellular overactivation and inflammatory cytokine release, further aggravating neuroinflammation. This immune network remodeling mechanistically illustrates the intricate interaction between the immune and nervous systems, potentially connecting to fundamental pathological processes of major depressive disorder-neurotransmitter imbalance, neuroinflammation, and neuroendocrine dysfunction-through pathways including cytokine release, microglial activation, and modified neuronal plasticity (37). This study has substantial clinical implications: immune network remodeling may function as a biomarker for the diagnosis and severity evaluation of MDD, while focused immunomodulatory therapies, such as immunomodulators and anti-inflammatory medications, offer intriguing new therapy options.

Within the single-cell sequencing dataset, we further identified 1,539 DEGs2. GSVA of DEGs2 revealed significant enrichment of the mammalian circadian rhythm pathway in NK cells from MDD samples. This suggests that immune system function, including NK cell activity, in MDD patients may be influenced by circadian rhythm disruption (38). Given the established link between MDD and immune dysfunction (39), this association could imply either heightened circadian regulation of NK cell activity in MDD or intrinsic dysregulation of the circadian pathway within NK cells. Furthermore, comparative analysis of metabolic pathway activity in NK cells between control and MDD groups identified significant differences in pathways including Ubiquinone and other terpenoid-quinone biosynthesis, Butanoate metabolism, Synthesis and degradation of ketone bodies, Terpenoid backbone biosynthesis, Galactose metabolism, and Folate biosynthesis. Ubiquinone, a crucial component of the mitochondrial electron transport chain, is essential for maintaining mitochondrial function (40). Given the high energy demands for NK cell activation and effector functions, mitochondrial integrity and ubiquinone-dependent oxidative phosphorylation are particularly critical (41). Reduced activity of this pathway in MDD may lead to cellular energy deficiency, impairing NK cell cytotoxicity and immunoregulatory functions (42). Butyrate, a major short-chain fatty acid (SCFA) produced by gut microbiota fermentation of dietary fiber, possesses potent anti-inflammatory and immunomodulatory properties capable of influencing various immune cells, including NK cells (43). Diminished butanoate metabolism activity in MDD patients could exacerbate gut inflammation, indirectly impairing NK cell function (44). Collectively, these metabolic pathway alterations reflect significant metabolic reprogramming in NK cells during MDD, impacting key aspects of energy metabolism, immune regulation, and cellular function, thereby providing novel insights for understanding NK cell roles in MDD pathogenesis and developing targeted immunometabolic therapies.

This study ultimately identified five key genes: CSPP1, ZNF84, HLA-DPA1, CCZ1, and LRRC8D. A logistic regression model constructed using these genes demonstrated robust predictive performance for MDD. CSPP1 (centrosome and spindle pole-associated protein 1) participates in microtubule stabilization and cell cycle regulation (with peak expression during G2/M phase), playing a vital role in maintaining cytoskeletal dynamics and ensuring precise chromosome segregation during mitosis (45–47) Its aberrant expression is recognized as a biomarker in various cancers (48). Zinc finger protein 84 (ZNF84) is a zinc finger-containing transcriptional regulator implicated in cell differentiation, proliferation, and tumorigenesis (49, 50). The HLA-DPA1 gene encodes a major histocompatibility complex (MHC) class II molecule, playing a central role in antigen presentation and the activation of CD4+ T cell-mediated adaptive immune responses (51–53). Quantitative Trait Locus (QTL) analyses and brain gene expression studies suggest that HLA-DPA1 may contribute to immune-related alterations in psychiatric disorders (53), indicating its genetic variation or expression levels might influence MDD susceptibility or severity. CCZ1 (homolog) is involved in vacuolar protein sorting (VPS), endosomal trafficking, and autophagy; its deficiency leads to abnormal vacuolar morphology (54, 55). Considering the observed reduction in autophagy levels in the brains of MDD patients and the modulatory effects of antidepressants on autophagy (56), CCZ1 dysfunction may contribute to MDD pathology by disrupting neuronal autophagic homeostasis. LRRC8D (leucine-rich repeat-containing 8D) is a component of the volume-regulated anion channel (VRAC), and its mutation or dysregulation is associated with drug resistance and metabolic disorders (57, 58). Research on these key genes (CSPP1, ZNF84, HLA-DPA1, CCZ1, LRRC8D) in the context of MDD remains limited. This study provides novel perspectives and potential biomarkers for understanding MDD molecular mechanisms and diagnosis. To further elucidate the molecular mechanisms of these key genes in MDD, Gene Set Enrichment Analysis (GSEA) was performed. This revealed that the key genes may modulate MDD through pathways such as Parkinson’s disease, oxidative phosphorylation, and Alzheimer’s disease. Clinical and epidemiological studies indicate a strong relationship between neurodegenerative disorders like Alzheimer’s disease (AD) and psychiatric conditions including MDD (59). Depression is a common and debilitating non-motor symptom in Parkinson’s disease (PD), significantly affecting quality of life (60), with studies suggesting up to 50% of PD patients experience depression during their disease course (61). Mitochondrial dysfunction plays a significant role in MDD pathophysiology. Studies on MDD patient brains reveal reduced activity of mitochondrial respiratory chain complexes, leading to decreased energy production, increased reactive oxygen species (ROS), and consequently, impaired neuronal function. The oxidative phosphorylation pathway involves several key enzymes whose abnormal activity may be linked to MDD. For instance, cytochrome c oxidase (complex IV), the terminal enzyme in this pathway, exhibits reduced activity in MDD, potentially causing electron transport chain blockade, diminished energy generation, and increased ROS production. Excessive ROS accumulation induces oxidative stress, damaging cellular structures and functions, including neurons (37).

To gain deeper mechanistic insights into the regulation of these key genes, we constructed competing endogenous RNA (ceRNA) regulatory networks and transcription factor (TF) regulatory networks. The ceRNA network operates through RNA molecules (e.g., lncRNAs, circRNAs) competitively binding microRNAs (miRNAs), thereby modulating target mRNA expression levels (62, 63). This study identified several potential ceRNA regulatory axes, including NEAT1/miR-545-5p/CCZ1, MALAT1/miR-205-5p/CSPP1, AC021078.1/miR-181d-5p/LRRC8D, and SNHG29/miR-513a-5p/ZNF84. Within the TF regulatory network, the key gene HLA-DPA1 exhibits regulatory relationships with transcription factors JUN, JUND, RUNX3, and NFKB1. The specific functional roles of these regulatory axes in MDD pathogenesis warrant further experimental validation. Screening for compounds targeting these key genes holds promise for developing novel targeted therapeutics for MDD.

This study identifies NK cell dysfunction as a core pathophysiological mechanism in MDD, characterized by aberrant cellular expansion and significant metabolic perturbations. The key genes (CSPP1, ZNF84, HLA-DPA1, CCZ1, and LRRC8D) identified in this investigation exhibit robust potential as diagnostic biomarkers and promising therapeutic targets for MDD. Furthermore, the elucidation of their underlying regulatory networks provides critical mechanistic insights to inform the development of precision-based interventions in psychiatry. Despite the limited scRNA-seq sample size (three cases and three controls), the integration of bulk transcriptomics, WGCNA, single-cell profiling, and pseudotime analysis provides cross-level validation and enhances the robustness of our findings. Nevertheless, we acknowledge this limitation, and future studies with larger cohorts and experimental validation will be required to further confirm the identified NK-cell alterations and gene regulatory mechanisms.

## Data Availability

The datasets presented in this study can be found in online repositories. The names of the repository/repositories and accession number(s) can be found below: https://www.ncbi.nlm.nih.gov/geo/, GSE39653.
