# Supplementary figures and images for: Multi-omics integration identifies NK cell dysregulation and a five-gene diagnostic signature in major depressive disorder
Source: Front Immunol. 2026 Jan 12;16:1700629. doi: 10.3389/fimmu.2025.1700629 (PMC12832902; doi:10.3389/fimmu.2025.1700629)

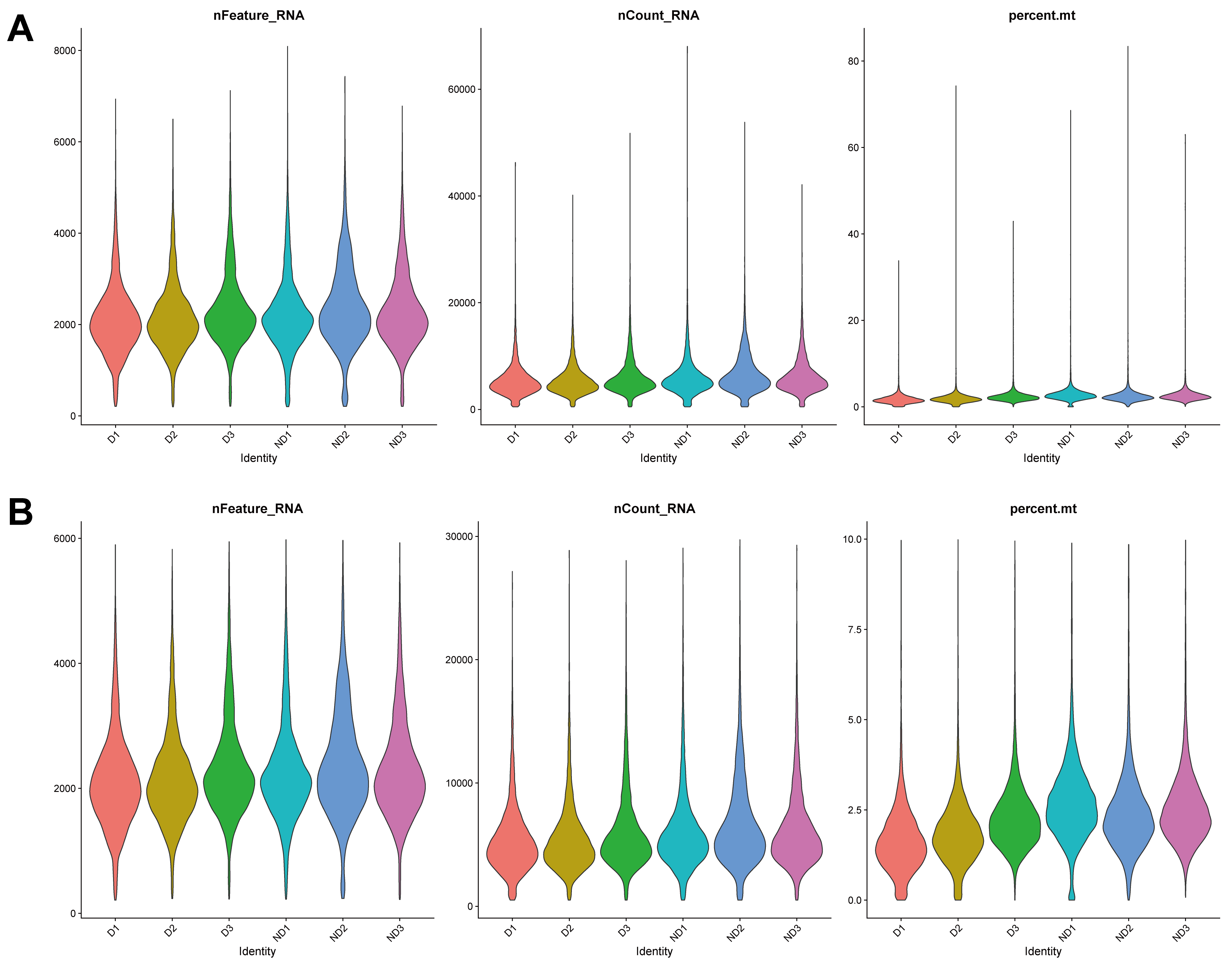

Supplement: Supplementary file 1 [file Image1.jpeg]
